# Phonological similarity between words is represented in declarative memory as spatial distance

**DOI:** 10.1007/s00426-023-01830-y

**Published:** 2023-05-19

**Authors:** Cosimo Tuena, Daniele Di Lernia, Giuseppe Riva, Silvia Serino, Claudia Repetto

**Affiliations:** 1 Applied Technology for Neuro-Psychology Lab, IRCCS Istituto Auxologico Italiano, Via Magnasco 2, 20149 Milan, Italy; 2 Humane Technology Lab, Università Cattolica del Sacro Cuore, Milan, Italy; 3 Present Address: Department of Psychology, Università degli Studi Milano – Bicocca, Piazza Dell’Ateneo Nuovo, 20126 Milan, Italy; 4 Department of Psychology, Catholic University of the Sacred Heart of Milan, Milan, Italy

## Abstract

Space can be used as a metaphor to describe semantic and perceptual similarity. Research has shown that similarity and spatial information can influence each other. On the one hand, similarity entails spatial closeness; on the other hand, proximity leads to similarity judgment. This spatial information can be stored in declarative memory and measured later on. However, it is unknown if phonological similarity/dissimilarity between words is represented as spatial closeness/distance in declarative memory. In this study, 61 young adults were tested on a remember-know (RK) spatial distance task. Participants learned noun pairs on the PC screen that were manipulated concerning their phonological similarity (similar vs. dissimilar) and reciprocal spatial distance (near vs. far). In the recognition phase, old-new, RK, and spatial distance judgments were asked. We found that for hit responses in both R and K judgments, phonologically similar word pairs were remembered closer compared to phonologically dissimilar pairs. This was also true for false alarms after K judgments. Lastly, the actual spatial distance at encoding was only retained for hit R responses. Results suggest that phonological similarity/dissimilarity is represented respectively with spatial closeness/distance and that this information is stored in the neurocognitive system of declarative memory.

**Supplementary Information:**

The online version contains supplementary material available at 10.1007/s00426-023-01830-y.

## Introduction

Similarity is an analogical process where perceptual (e.g., sounds) or abstract (e.g., political ideologies) properties can be described using a metaphor. Space is often used to support such analogies as people frequently use the terms ‘close’ and ‘far apart’ to describe similar and dissimilar things (Casasanto, 2008). In addition, in accordance with the construal level theory (Trope & Liberman, 2010), space, in terms of psychological distance, helps us to transcend the immediate situation and to represent things not directly accessible in the current experience (e.g., memories, plans, prediction, hopes, and counterfactual alternatives).

Understanding how similarity is structured and organized as a cognitive representation is one of the questions of psychological and cognitive science (Decock & Douven, 2011). Lakoff & Johnson, (1999) proposed two complementary conceptual mappings to describe the tight link between space and similarity: spatial closeness as similarity and spatial distance as dissimilarity or alternatively, similarity as spatial closeness and dissimilarity as spatial distance. According to the conceptual metaphor theory (Lakoff & Johnson, 1980, 1999), these metaphors are grounded in our sensorimotor experience with the world. Embodied and grounded cognition theories support the notion that conceptual metaphors and knowledge are rooted in our experience with the body and environment, that is to say, that the domains of space, force, or motion, which are experienced through our sensorimotor system, are used to describe abstract concepts and create metaphors (Barsalou, 1999, 2008; Gallese & Lakoff, 2005; Wilson, 2002).

A consistent body of evidence suggests the importance of the space for the representation of similarity, particularly within the semantic domain (i.e., the conceptual metaphor direction tested here is ‘proximity → similarity’). In one seminal study, Casasanto, (2008) presented participants with a series of item pairs (abstract nouns, faces, object pictures) at different distances on a computer screen, and then they were asked to judge how much the items were similar in their meaning (abstract nouns), functional use (objects), or visual appearance (objects and faces). Results indicated that abstract nouns and object pictures (functional use) pairs were rated as more similar when they were presented in the near space (as opposed to the far space); the opposite pattern was found for face pairs and object pictures (visual appearance). The author concluded that when participants were asked to make conceptual judgments (namely, when they were asked to make judgments about the meaning of nouns and the functional use of objects), the spatial proximity influenced similarity judgments; conversely, when participants were asked to make perceptual judgments, the spatial proximity operates in the opposite direction. Guerra & Knoeferle, (2014) showed that spatial proximity can affect subsequent abstract sentence comprehension of semantically related nouns. When noun pairs (e.g., ‘joy’ and ‘euphory’) were presented near on the PC screen and the sentence in which they were contextualized expressed similarity (“*Joy and euphory are almost similar*”), participants’ reading times at the adjective (i.e., ‘similar’) were faster compared to the condition in which the nouns were far; conversely, when noun pairs were presented far apart compared to the condition in which they were close, participants’ reading times at the adjective were faster for sentences that conveyed dissimilarity.

Conversely, other evidence has been provided in support of the opposite direction of the metaphor (i.e., ‘similarity → proximity’). Boot & Pecher, (2010) showed that participants were faster to make judgments on colored squares when they were similar in color and near, compared to the condition when the squares were far; in addition, they were faster to respond when the squares were far and dissimilar in color compared to near. They also found that the relation between similarity and closeness is asymmetrical, supporting the notion that similarity entails the spatial properties of closeness, but not the other way round.

Interestingly, grounded knowledge and abstract concepts are organized within cognitive architectures, namely *low-dimensional spaces* (Bottini & Doeller, 2020). Here, information is conceptualized as points in a space that is formed by a few characteristics of the information (e.g., jobs can be described depending on ‘freedom’ and ‘loneliness’ axes; Bottini & Doeller, 2020). Research shows that items in these architectures are represented through an abstract spatial distance (Theves et al., 2019). Solomon et al., 2019 showed that the abstract distance computation is relevant for words stored in the declarative memory system. Distances were measured as semantic distance, computed from Euclidean distance in word2vec subspaces and as temporal distances, calculated from the serial position of the word at retrieval. The authors showed that items are stored in episodic memory according to their temporal and, particularly, semantic distance. They concluded that declarative memory is supported by item associations computed as distances in an abstract cognitive space.

Semantic aspects of language have been investigated by multiple studies; however, less is known regarding how other language characteristics are organized in terms of space. In the current study, we focused on phonology, namely the abstract representation of speech sounds in a certain language. There is growing evidence that phonology is created, stored, and shared through the embodied learning of sound production, shaped by the inputs in the environment but also by the experience of creating sounds and recognizing them (Fogassi & Ferrari, 2008; Nathan, 2017). Berent & Platt, (2022) showed phonetics and lexical phonological associations are grounded in our sensorimotor system, whereas the phonological structure is abstract. These perceptual aspects of phonology were found to hamper memory performance. Early studies on the so-called phonological similarity effect showed that the effect occurs as a result of interference in the phonological store between similar phonological memory traces (Baddeley, 1986). In this sense, the immediate serial recall of phonologically similar words is poorer compared to phonologically dissimilar words. Conversely, other recent findings suggest that phonological similarity improves memory recall independently from the method used to test retrieval of the items (Gupta et al., 2005). Phonological similarity also affects recognition memory and could vary depending on the language (e.g., Chan & Vitevitch, 2009; Luce & Pisoni, 1998; Vitevitch & Rodríguez, 2005). For instance, Spanish words with high frequency of similar words (i.e., neighborhood) are recognized more quickly and accurately than words with low neighborhood frequency, whereas the opposite result is found in English. As a consequence of these effects on memory, phonology can affect word learning by improving phonological processing (e.g., directing attention toward phonological aspects of words) and pronounceability at encoding or be influenced by phonological similarity (e.g., neighborhood density) or phonological skills (Meade, 2020; Stamer & Vitevitch, 2012).

Despite many studies being done regarding the effect of phonology on memory, to our knowledge, there is no study concerning how words stored in declarative memory are spatially organized depending on their phonological similarity. Particularly, no study has investigated if the abstract concept of space is used offline (stored in memory) to represent words that are close or far apart in terms of phonological characteristics.

To pursue this aim, we developed an old-new recognition test followed by remember-know (RK; Migo et al., 2012; Wixted & Stretch, 2004) and spatial distance judgments. We applied the RK procedure to assess the two domains of declarative memory, respectively episodic and semantic memory (Migo et al., 2012; Wixted & Stretch, 2004). In the encoding phase, participants were presented with noun pairs that were manipulated according to two dimensions: reciprocal spatial location (far-near) and phonological similarity (alliterative-dissimilar). Then during the recognition phase, participants completed an old-new recognition memory test. If participants answered ‘old’, RK judgments were asked followed by the spatial distance estimation task.

In this study we wanted to explore if: (1) regardless of the actual encoding spatial location, phonological characteristics of the word pairs will affect subsequent recalled spatial distance (similar words are judged closer and dissimilar words farther); (2) actual and phonological spatial distance estimation of word pairs is influenced depending on the declarative memory (episodic memory vs. semantic, R vs. K respectively) system in which this information is stored; (3) phonological characteristics of the word pairs will affect false alarms (FA) in the same way as explained in the first point (i.e., even when not spatially presented at encoding, similar word pairs are represented closer and dissimilar pairs farther apart). In conclusion, we expect that in addition to physical spatial properties (i.e., actual spatial location) phonological characteristics of words retain abstract spatial information that can be stored in memory as the phonological spatial distance between dissimilar and similar noun pairs.

## Methods

### Participants

Sixty-one healthy young adults were recruited for this study (*M*_age_ = 23.25, SD_age_ = 4.04, *M*_edu_ = 14.87, SD_edu_ = 2.76; females = 25; right-handed = 56). Participants were recruited online via Prolific (https://app.prolific.co/) due to the COVID-19 pandemic restrictions during December 2021. Participants were paid 8.73 € per hour (the experiment lasted 20 min approx.; range = 13m55sec–22m53sec). Inclusion criteria were Italian as a native language and normal-to-correct vision. Exclusion criteria were self-reported language-related disorders, literacy difficulties, history of head injury, cognitive deficits, amnesia, long-term/chronic disabilities, and psychiatric medications. With a Cohen’s d of 0.33, a power of 0.8, 60 stimuli, and a fully crossed design, the power analysis for a mixed-effects model (Westfall et al., 2014) required a minimum of 59 participants. Cohen’s d was extracted from the difference between dissimilar and alliterative words at recall (Gupta et al., 2005). Participants gave their consent to participate before the experiment began. The study was approved by the Ethical Committee of the Catholic University of Milan.

### Stimuli

One hundred two-syllable nouns (with four or five letters) were selected for the experiment. We used two-syllable nouns because in Italian this is the minimum number of syllables needed to obtain a pool of words large enough for the present task. Part of these words (*N* = 84) was taken from Montefinese et al., (2014) database, whereas the remaining (*N* = 16) were added by the authors and validated using the same dimensions (emotional valence, familiarity, concreteness, and imageability). The validation study was administered to 21 adults. Averaged values from the participants for each word were calculated and any outliers were removed. T-tests were carried out to find differences between the dissimilar and alliterative groups and yielded no differences for any dimensions (emotional valence, familiarity, concreteness, and imageability) between the two groups. In addition, we evaluated, with the CoLFIS database (Bertinetto et al., 2005), the lexical frequency (average frequency for each word pair). We found no difference (t-test) between alliterative and dissimilar phonology groups.

Alliterative words (the first two phonemes between two words overlap, Gupta et al., 2005; i.e., ‘no-ce’/‘no-do’; ‘nut’/’knot’) were considered similar in phonology to each other, whereas words without the same sound on the first two phonemes were considered dissimilar (‘fi-ore/to-po’; ‘flower’/’rat’). Words were randomly paired to create alliterative and dissimilar noun pairs. We used alliterative phonemes given their intermediate effect on memory recall (Gupta et al., 2005). In their five experiments (Gupta et al., 2005), the authors consistently found that rhyming word lists (i.e., mat, fat, sat, rat, hat, bat) led to better serial recall performances compared to alliterative word lists (i.e., cat, cab, cad, can, cap), and canonically similar word lists (i.e., cad, cat, map, can, man). These findings (i.e., rhyming > alliterative > canonical) are consistent with a serial order account. This view proposes that words that share the same phoneme at the end are easier to produce compared to alliterative words, which, in turn, are easier to produce than canonically similar words (Gupta & Dell, 1999; Gupta et al., 2005). Finally, we evaluated semantic similarity by calculating the Wu & Palmer, (1994) index with Python WordNet (the index ranges from 0—no similarity—to 1—maximum of similarity—and determines relatedness by taking into account the words synsets’ depths in the WordNet taxonomies). We found no difference (*p* > 0.05) in the index between alliterative and dissimilar phonology groups. In the end, the only aspect of the pairs that could make them similar/dissimilar was the phonology.

### RK spatial distance task

The task was designed thanks to the Gorilla platform for online behavioral experiments (Anwyl-Irvine et al., 2020). The task was divided into the encoding and an immediate recognition phase; the latter phase was divided into the old-new recognition part, the RK judgment task, and the spatial distance judgment task. Forty noun pairs (80 single words) were presented in the encoding phase; additional 20 noun pairs (40 total single words) were used as new items for the recall phase (see Supplementary Material 1 for item pairs in each condition). The old items were divided into 20 phonologically dissimilar and 20 alliterative noun pairs, similarly, the new items were further categorized into 10 phonologically dissimilar and 10 alliterative noun pairs. In the encoding phase, the phonologically dissimilar and alliterative old noun pairs were further divided into far and near spatial locations (i.e., 10 near-dissimilar, 10 far-dissimilar, 10 near-alliterative, 10 far-alliterative noun pairs). This resulted in four experimental conditions as in a similar study on similarity (Boot & Pecher, 2010; Guerra & Knoeferle, 2014). The near and far locations were defined in the following way. For each noun pair, we randomly chose a noun as ‘fixed’ and the other as ‘moving’ (i.e., located in variable positions across trials). The fixed noun was placed at the center of the bottom section of the PC screen. The moving noun was placed in line with the fixed noun and on the same vertical axis and across trials, was displayed at different distances from the associated fixed word. Using the fixation cross, the near and far portions were extracted. So that if the moving noun was below the fixation cross, it was considered near to the fixed word, conversely if the moving noun was displayed above the fixation cross, it was considered far from the fixed noun. The moving noun of each noun pair was randomly allocated to the far or near portion and this order was counterbalanced among the participants so that if the noun ‘noce’ (fixed on the vertical axis bottom location) was paired with the moving word ‘nodo’ in the near portion; for the next participant the noun ‘noce’ was kept fixed at the same location as described above but the moving noun ‘nodo’ was counterbalanced and moved to the correspondent far slot of the screen. The noun pairs were presented one at a time for 5 s, followed by a 1 s fixation cross that exactly divided the near and far portions of the screen.

**Fig. 1:**
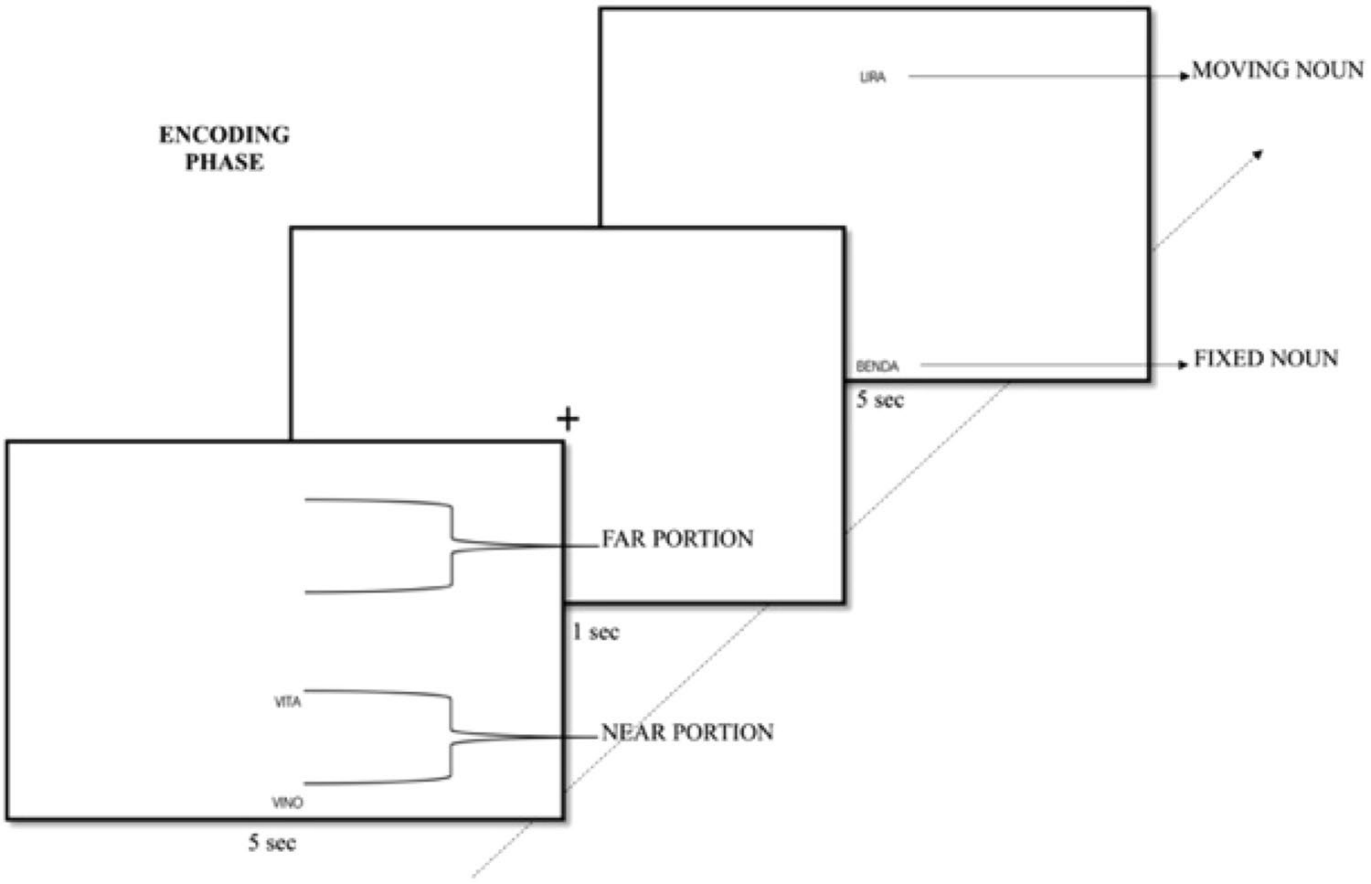
In the encoding phase, the moving noun of each word pair moved between near and far portions across the trials

In the encoding phase, participants were instructed to pay attention and memorize the word pairs (intentional encoding), the implicit task was therefore the encoding of the spatial locations of each noun pair. Pairs were randomly presented two times during this phase to improve encoding, as the objective of this study is to maximize the number of retained items for the recognition part. See Fig. 1 for the encoding phase flow.

**Fig. 2:**
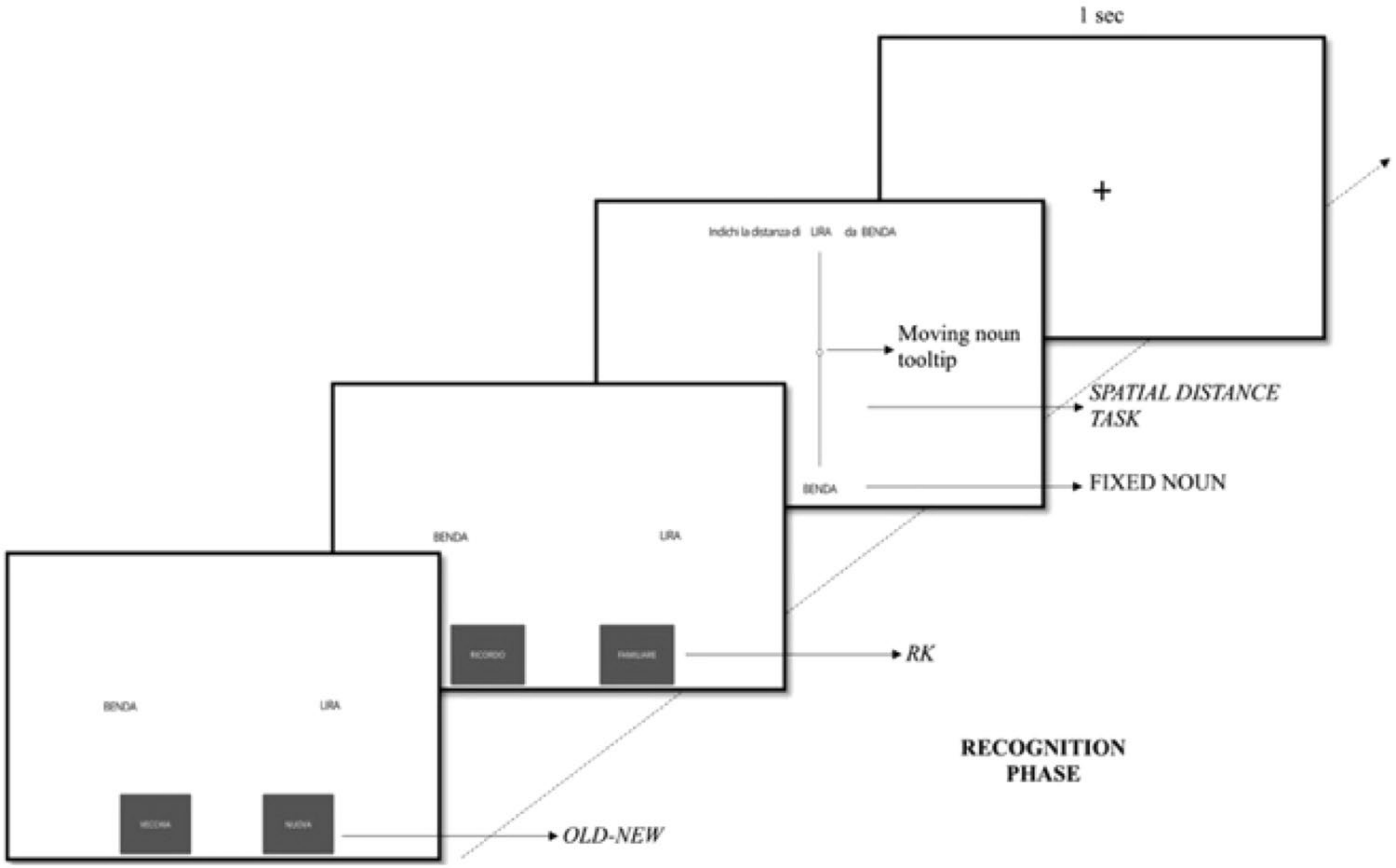
Procedure of the remember-know (RK) spatial distance judgment task. In the recognition phase, old-new, RK, and spatial distance tasks were performed

In the immediate recognition phase, all the old noun pairs and unlearned new item pairs were randomly presented to each participant. The noun pairs were shown on the horizontal axis equidistantly from the fixation cross to avoid any bias regarding the encoded vertical position. Participants were instructed to judge the noun pairs as old or new. If they rated the words as old, they were asked if they ‘remember’ or ‘know’ the noun pairs. For the old-new and RK responses, a 2-forced choice method was applied (no time limit to respond), and participants used the mouse to select the preferred box shown on the PC screen. After both R and K responses, they had to judge the distance of the moving noun from the fixed one. They could move the slider tooltip (i.e., position marker) with the mouse and once happy with the slider tooltip position, they pressed the spacebar to proceed to the following noun pair (no time limit to respond). In particular, the participant was presented with a vertical slider (underlying not visible values 0 to 5 by 0.1) covering the distance between the near and far portions, where the 2.5 slider value corresponded to the fixation cross. The starting position of the slider tooltip was in the middle of the slider range (i.e., the location of the fixation cross). The fixed word appeared at the same location as in the encoding phase. The participant was asked to move the slider tooltip to indicate where the second word was located during the encoding phase. Importantly, the spatial judgment task was unexpected as participants were instructed only to memorize the word pairs and not their spatial location on the PC screen. Lastly, if the participant responded ‘new’, the following noun pair was presented, skipping the RK section of the task (and consequently the spatial judgment task). A 1 s fixation cross was presented after each new response or spatial distance judgment response. See Fig. 2 for the immediate recognition phase procedure. In addition, 13 total attentional checks (“*press the box with the number 1*” –button box ‘1’ or button box ‘10’ choices) were put across the encoding and recognition phases according to suggestions provided by Gorilla (Anwyl-Irvine et al., 2020).

### Procedure

The experiment was conducted online due to the COVID-19 pandemic. Once the experiment was published on the Prolific system, participants meeting the preselected inclusion/exclusion criteria could access the Gorilla link to start the experiment. After ticking the consent form (mandatory for proceeding further), demographical (i.e., age, sex, education, dominant hand) information was collected. After that, participants read the following instructions: “*Now you will see some word pairs. Your task is to memorize the word pairs. Pay attention because each pair of words will only be visible for a few seconds. You will see each pair of words twice in random order throughout the presentation. The word pairs will be shown automatically*”. After the encoding phase, the recognition phase instructions were displayed: “*Now you will see some word pairs and you will have to indicate if: the pair is old, that is, if you saw it among the pairs of words presented before (‘OLD’ button); the pair is new, that is to say, that you have not seen it among the pairs of words presented before (‘NEW’ button. If you answer ‘OLD’, you will be asked if: you remember the pair of words, that is, if you have a detailed memory of the noun pair (‘REMEMBER’ button); the pair is familiar to you, i.e., if you know you have seen them but do not have a detailed memory of it (‘KNOW’ button). Once indicated if you remember/know the word pair, you will also have to indicate the distance of the words using a slider*”. During this latter phase on the top section of the screen of each spatial judgment, the participant is prompted to indicate the distance of the moving from the fixed noun (the actual nouns are presented, e.g., “*indicate the distance of NODO from NOCE”*). Any questions or technical issues could be resolved thanks to the Prolific chat with the principal investigator of the experiment.

### Statistical analyses and data pre-processing

Statistical analyses were carried out with R (version 3.6.3) (R Core Team, 2014). Linear mixed-effect model (LMM) ANOVA using Satterthwaite approximation was used in the analyses (Luke, 2017). Bobyqa optimizer was applied to ensure model convergence (Brown, 2021). Models were specified to have a random intercept for noun pairs and participants, as random intercept and slope models failed to converge [formula = outcome ~ fixed effects + covariate + (1|participant) + (1|noun pair)]. The fixed effects were the encoding spatial location (near-far) and the phonological similarity (alliterative-dissimilar) of the noun pairs. To control for different computer screen heights used by the participants that could bias spatial encoding and recalled spatial distance we put height in pixels (px) recorded by Gorilla software as a covariate in each model. This resulted in a 2 × 2 within-subjects ANCOVA. Variance explained by random effects on the dependent variable was provided by the intraclass correlation coefficient (ICC). *lme4* R package was used to run the LMM analyses (Bates et al., 2015). LMM assumptions of normality of residuals and homoscedasticity were verified by visual inspection. Partial eta squared (*η*^2^_*p*_) was interpreted (small = 0.01, medium = 0.06, and large = 0.14) according to Richardson (2011). To test the amount of evidence for our findings, we also used Bayesian statistics. Bayes factor bound (BFB) computation was carried out as suggested to improve p-value interpretation (Benjamin & Berger, 2019). Jeffreys’s rule of thumb for BFB interpretation was used (Ly et al., 2016). Evidence from the data in favor of H1 relative to H0 (i.e., BFB), odds in favor of H1 relative to H0, and ‘post-experimental odds’ combined with prior odds of H1 to H0 (prior odds were set to 1:1) were computed as suggested (Benjamin & Berger, 2019).

Regarding pre-processing, to ensure that participants were providing above-chance responses in the recognition phase, d-prime (*d*’) was used to find participants with as-chance performance (i.e., *d*’ = 0) (Green & Swets, 1966). Two participants were found to have random guessing performances. These were excluded from all the analyses as we could not rule out the possibility that they were carrying out the online task with due motivation. Responses of the old-new, RK, and spatial judgment tasks exceeding 500 ms or 5000 ms were excluded and coded as missing values that can be handled properly by LMM (Brown, 2021). Correct rejection (CR) had no missing values out of 1029 responses, miss had 38 out of 544 responses coded as missing values. Regarding RK FA, 20/123 responses were coded as missing values and regarding the spatial distance judgment task 10/123 were coded as missing. Concerning R hit, 293/1209 responses were coded as missing values and regarding the spatial distance judgment task 133/1209 were coded as missing; for K hit, 75/607 responses were coded as missing values and regarding the spatial distance judgment task 54/607 were coded as missing. All the participants responded correctly to the attentional checks (range 12–13 out of 13). Values in the graphs and result section are the predicted values of the LMM. The significance level for all the analyses was set to 0.05.

## Results

### Recognition accuracy performance

The average recognition memory performance (n° hit/40) across the participants and conditions was 77% (SD = 23.13). Table 1 shows the old-new performances by the condition in detail.

**Table 1:** Average recognition memory and spatial distance task accuracy

Old-new task accuracy performance				
Response	Far-alliterative	Far-dissimilar	Near-alliterative	Near-dissimilar
Hit	7.83 (2.26)	7.42 (2.53)	7.88 (2.11)	7.64 (2.37)
Miss	2.92 (2.13)	3.64 (2.17)	3.05 (1.86)	3.2 (2.1)

Response	Alliterative	Dissimilar
CR	9.31 (1.18)	8.14 (2.14)
FA	2.85 (1.72)	2.07 (1.27)

Spatial distance task accuracy performance				
Response	Far-alliterative	Far-dissimilar	Near-alliterative	Near-dissimilar
R correct spatial recall	156/267 (missing = 30)	175/268 (missing = 37)	217/277 (missing = 27)	189/274 (missing = 29)
K correct spatial recall	70/151 (missing = 14)	72/121 (missing = 12)	0/143 (missing = 18)	0/138 (missing = 10)

In the old-new section mean and SD are reported. CR and FA do not have spatial encoding positions because were not shown in the encoding phase of the task. Missing are responses exceeding 500 ms and 5000 ms

*CR* correct rejection; *FA* false alarms; *R* Remember; *K* know

The accuracy of the spatial distance task was extracted in the following way. The values between 0 and 2.49 represent the near portion, whereas values between 2.51 and 5 the far portion (2.5 is the position of the fixation cross). If the participants put the slider tooltip in the near portion and the moving noun at encoding was in the near to the fixed noun it was coded as correct, if the participants put the slider tooltip in the far portion and the moving noun at encoding was in the far section of the screen relative to the fixed noun position again it was coded as correct. Conversely, if the slider tooltip was placed in the opposite portion of the screen, responses were recorded as incorrect (e.g., if the slider tooltip was placed above the fixation cross—far portion—but at encoding the moving noun was below the cross—near to the fixed noun—the response was incorrect). Table 1 shows the spatial distance performances by condition.

Regarding the d’ on the old-new responses depending on the encoding spatial location (near-far) and noun pairs phonology (dissimilar-alliterative), we found only a main effect of phonology (*F*_1_ = 39.83, *p* < 0.001, *η*^2^_*p*_ = 0.19, 95%CI [0.09, 0.29]). Higher recognition accuracy for dissimilar (*M* = 2.26, SE = 0.09) than alliterative word pairs (*M* = 1.93, SE = 0.1) was found. No effect of encoding spatial location or interaction effect was found. Importantly, the average d’ for the four conditions exceeded the cut-off (*d*’ = 0) of random guessing. The d’ for the far-alliterative condition was 1.92 (SD = 1.04), for the far-dissimilar was 2.24 (SD = 0.96), for the near-alliterative was 1.94 (SD = 1.09), and for the near-dissimilar was 2.29 (SD = 0.94).

### Spatial distance task performance

LMM ANCOVAs were used to analyze the impact of spatial distance (near-far) and phonology (dissimilar-alliterative) conditions at encoding on the recalled spatial distance while controlling for computer screen height (px). Separate within LMM ANCOVAs (word phonology: 2 levels; encoding spatial location: 2 levels; screen height as a covariate) were used to analyze R and K responses.

**Fig. 3:**
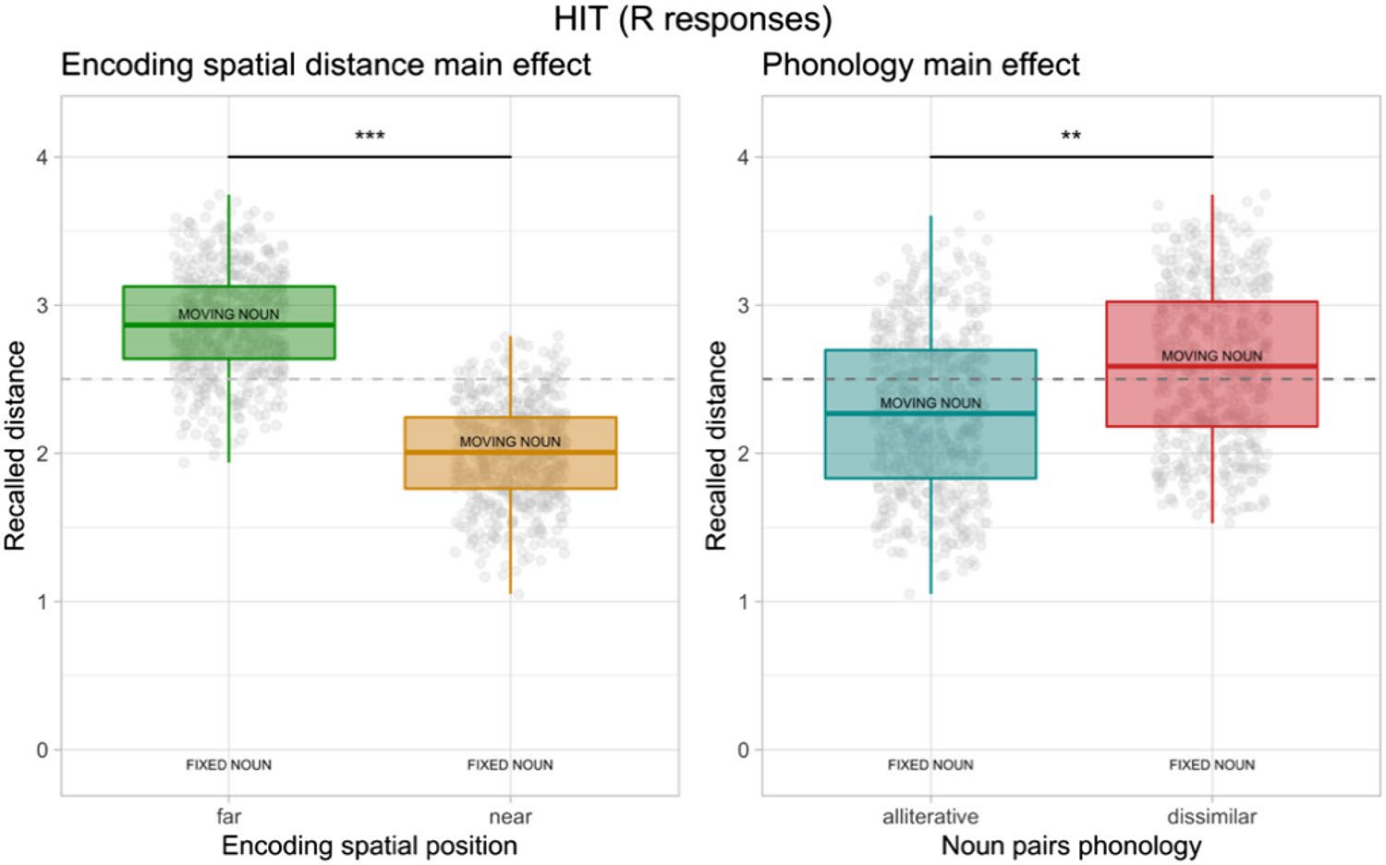
Spatial distance judgments results for the hits after remember (R) responses. The dashed line represents the boundary of the near and far portions of the screen relative to the fixed noun (i.e., fixation cross position). Boxplots depict the recalled distance (range 0–5) of the moving from the fixed word during the spatial distance task. ** < 0.01; *** < 0.001

Concerning the spatial distance judgments of R responses, ICC for the random effects was 0.07. We found a significant effect of the encoding spatial location (*F*_1_ = 121.66, *p* < 0.001, *η*^2^_*p*_ = 0.11, 95%CI [0.07,0.14]) and a significant main effect of phonology (*F*_1_ = 8.23, *p* = 0.007, *η*^2^_*p*_ = 0.18, 95%CI [0.02,0.39]). A significant effect of the covariate screen height was found (*F*_1_ = 4.97, *p* = 0.031, *η*^2^_*p*_ = 0.11, 95%CI [0.00,0.31]). Regarding the main effect of encoding spatial distance, noun pairs that were encoded as near were recalled closer (*M* = 2, SE = 0.01) compared to noun pairs that were encoded as far (*M* = 2.88, SE = 0.01). Regarding the main effect of noun pairs phonology, alliterative noun pairs are recalled closer (*M* = 2.28, SE = 0.02) than the dissimilar noun pairs (*M* = 2.59, SE = 0.02). Regarding the covariate, the higher the screen the shorter the recalled spatial distance. No interaction effect between phonology and spatial location was found. See Fig. 3 for these main effect findings.

**Fig. 4:**
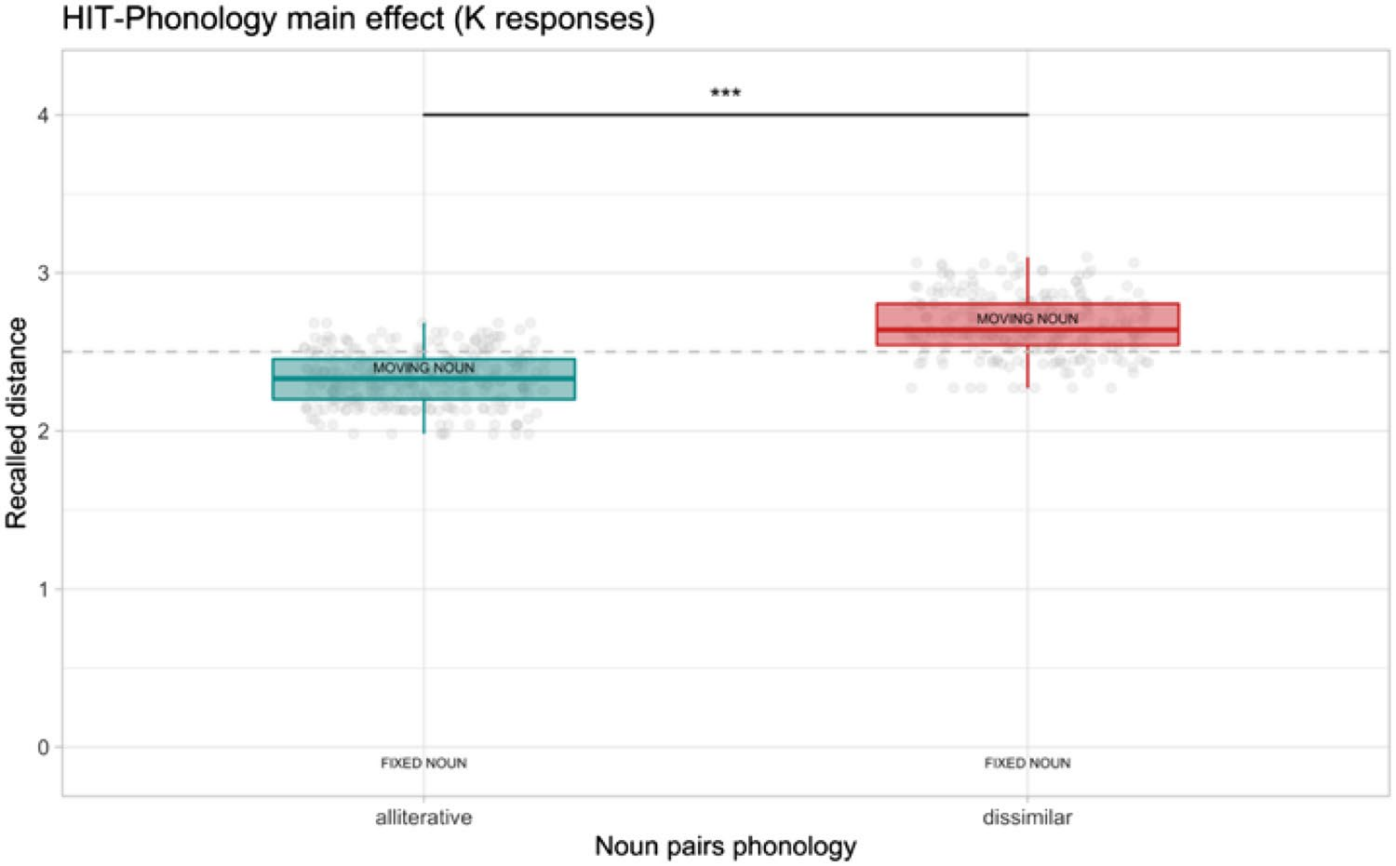
Spatial distance judgments result for hits after know (K) responses. The dashed line represents the boundary of the near and far portions of the screen relative to the fixed noun (i.e., fixation cross position). Boxplots depict the recalled distance (range 0–5) of the moving from the fixed word during the spatial distance task. *** < 0.001

Regarding the spatial distance judgments of K responses, ICC for the random effects was 0.04. We found only a significant effect of phonology (*F*_1_ = 13.7, *p* < 0.001, *η*^2^_*p*_ = 0.03, 95%CI [0.01,0.06]). Alliterative word pairs were recalled closer (*M* = 2.33, SE = 0.01) compared to dissimilar noun pairs (*M* = 2.67, SE = 0.01). Again, no interaction effect between phonology and spatial location was found. See Fig. 4 for the results of K responses.

**Fig. 5:**
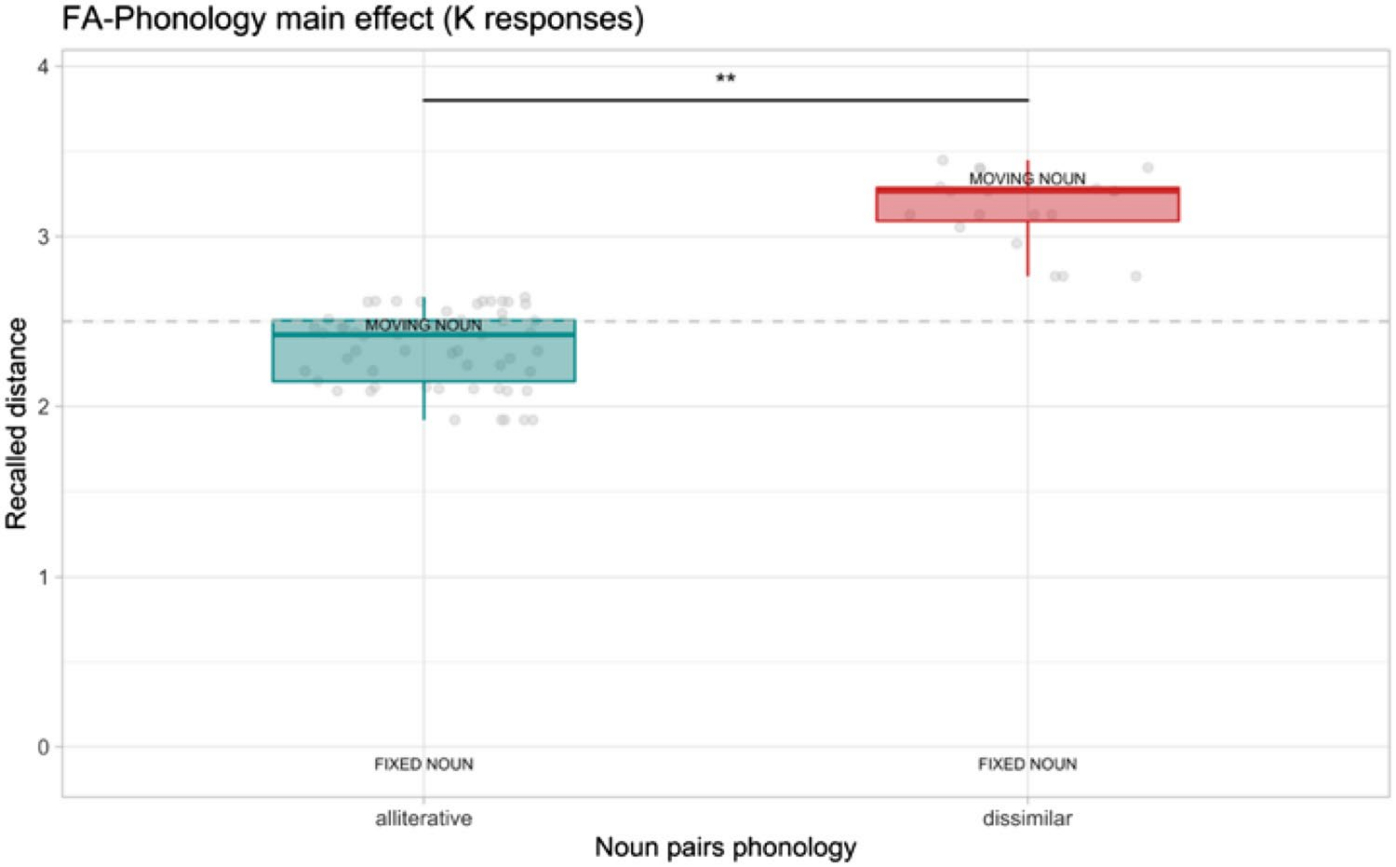
Spatial distance judgments result for false alarms (FA) after the know (K) responses. The dashed line represents the boundary of the near and far portions of the screen relative to the fixed noun (i.e., fixation cross position). Boxplots depict the recalled distance (range 0–5) of the moving from the fixed word during the spatial distance task. ** < 0.01

As a second step, we considered the FA. Concerning the spatial distance estimation after R, only 18 (six for dissimilar pairs) responses were given by the participants and the observed power of the LMM ANCOVA was 10%. Hence, analyses were not carried out. Regarding the spatial judgments after K, ICC for the random effects was 0.08. We found a significant effect of the phonology of nouns (*F*_1_ = 9.32, *p* = 0.003, *η*^2^_p_ = 0.11, 95%CI [0.01,0.26]). Alliterative noun pairs were judged to be closer (*M* = 2.34, SE = 0.03) compared to the dissimilar noun pairs (*M* = 3.16, SE = 0.05). The screen height covariate was not significant as participants did not see the word pairs in the encoding phase. See Fig. 5 for this result.

### Bayesian evidence of phonological distance in memory

To support our hypotheses, we performed Bayesian computation to improve p-value interpretation (Benjamin & Berger, 2019). Table 2 shows the amount of evidence from our results. Evidence from the data in favor of H1 (*μ*_phonological dissimilar_ ≠ *μ*_alliterative noun pairs_) relative to H0 (*μ*_phonological dissimilar_ = *μ*_alliterative noun pairs_) ranges from 21.17 to 186.38 (i.e., strong to extreme evidence; Ly et al., 2016). Odds in favor of H1 relative to H0 range from 0.91 to 0.99 (i.e., the probability of H0 being true ranges from 9 to 1%). Post-experimental odds with prior odds set to 1:1 (H1:H0) are in favor of H1 relative to H0 (see Table 2). The results show that an abstract phonological spatial distance between words exists, and this is particularly evident for K hit responses.

**Table 2:** Bayesian evidence in favor of an abstract spatial distance between noun pairs

Phonological distance	*p*-value	BFB	Pr^*U*^ (*H*_1_|*p*)	Post-experimental odds
HIT R	0.007	10.62	0.91	10:1
HIT K	< 0.001	> 186.38	0.99	> 186:1
FA K	0.003	21.17	0.95	21:1

Bayes factor bound (BFB) between 10 and 30 is indicative of strong evidence, BFB > 100 of extreme evidence; Pr^U^ (H_1_|p) is odds in favor of H1 relative to H0. Prior odds for the post-experimental odds were set at 1:1

## Discussion

In this study, we sought to explore if phonological similarity/dissimilarity between stimuli pairs is stored in memory as spatial proximity/distance, namely if perceptual representation of language entails the conceptual metaphor of space, where dissimilar characteristics are far apart and similar characteristics are near (Lakoff & Johnson, 1999). Our results indicate that alliterative word pairs are remembered closer than dissimilar word pairs, confirming our first hypothesis that, regardless of the actual encoded spatial location, phonological characteristics of the word pairs could affect subsequent recalled spatial distance. Our second hypothesis was that actual and phonological spatial distance estimation of word pairs is influenced by the declarative memory system in which this information is stored. In this regard, our results indicate that when information is stored in the episodic memory system (R responses), both encoded spatial information and phonological distance are preserved (i.e., word pairs encoded near and far are recalled near and far respectively but at the same time alliterative word pairs are recalled closer than dissimilar word pairs). Conversely, when information is stored in the semantic memory system (K responses), the spatial distance estimation is solely driven by the phonological characteristic of the word pairs (i.e., alliterative word pairs are recalled closer than dissimilar word pairs). On our third hypothesis, we sought to find if phonological characteristics (i.e., similar word pairs are believed to be closer than dissimilar word pairs even when not spatially presented at encoding) of the word pairs could affect false memories (i.e., FA). We showed that similar word pairs are represented closer and dissimilar pairs farther apart even when not spatially presented at encoding. However, this finding is especially true for false memories based on semantic features (K judgments).

These three results (main effect of phonology on distance estimation for hit R, hit K, and FA K) taken together show that, in addition to a remembered physical distance between words (main effect of encoding spatial distance for hit R), an abstract spatial distance exists and depends on the phonological similarity between the stimuli. Bayesian evidence demonstrates strong evidence of this abstract spatial distance (in favor of hit R and FA K responses and extreme evidence in favor of hit K judgments). The effect of phonological similarity of distance judgments is stronger for the latter result, however, we showed that this effect can influence spatial judgments after R and FA responses.

Our findings regarding the main effect of phonology on spatial distance estimation (first hypothesis) for hit R, hit K, and FA K responses extend previous theoretical and experimental studies. We showed that phonological aspects of language (in Italian) can support metaphorical conceptualization (see, Lakoff & Johnson, 1999). Different studies showed that proximity can be conceptualized as similarity and distance as dissimilarity (Casasanto, 2008; Guerra & Knoeferle, 2014), however, the opposite pattern is also true (Boot & Pecher, 2010). Previous research showed that spatial distance as a metaphor can be applied to semantic and perceptual materials (Boot & Pecher, 2010; Casasanto, 2008; Guerra & Knoeferle, 2014; Schneider & Mattes, 2021). In the study of Casasanto (2008), the author found that during conceptual judgments spatial proximity influenced similarity judgments; conversely, the spatial proximity operates in the opposite direction for perceptual judgments. That is to say that spatial proximity or distance leads to rate two items as closer or farther in terms of semantics (e.g., meaning of words), whereas spatial proximity or distance affects the judgments in the opposite direction for perceptual (e.g., object pictures) stimuli. Hence, Casasanto (2008) stated that physical closeness facilitates perceptual differences (latter case) or encourages semantic categorization (former case). We extend this finding because we showed that, regardless of the encoding spatial distance (i.e., no interaction between space at encoding and phonology), perceptual (e.g., potentially embodied) aspects of phonology affect the spatial distance estimation. This could suggest that there are inherently conceptual embodied representations of phonology, unrelated to physical distance, that are linked to the abstract concept of space as an index of similarity/dissimilarity. Indeed, Boot & Pecher, (2010), using perceptual stimuli, found that perceptual similarity leads to spatial closeness judgments. This is in line with our results, where phonological similarity/dissimilarity affected the recalled spatial distance. Nevertheless, we must acknowledge that the design of this experiment does not directly test a particular direction (‘similarity → proximity’ or ‘proximity → similarity’) of the conceptual mappings proposed by Lakoff & Johnson, (1999) between space and similarity and this remains an open question.

Previous research showed that phonology affects learning, working memory recall, and recognition of word lists (Gupta et al., 2005; Luce & Pisoni, 1998; Stamer & Vitevitch, 2012). Here, we found that phonological similarity affects spatial judgments after both episodic and semantic recognition (second hypothesis). Indeed, the serial parallel independent model (Tulving, 2001; Tulving & Markowitsch, 1998) states that perceptual (i.e., the phonological representation of the items), semantic (i.e., whether item pairs are phonologically similar or not), and episodic (i.e., items spatial location) information is encoded serially, stored in parallel, and the retrieval is independent and can entail other systems information. The retrieval of episodic information (R responses) includes perceptual, semantic, and episodic information, whereas semantic information (K responses) includes only perceptual and semantic characteristics of the items. Hence, the phonological distance is present in both R and K responses, whereas the spatial information can be accessed only for R responses and independently from other systems.

We also showed that this abstract conceptualization of distance between noun pairs is retained in long-term memory and particularly in episodic and semantic memory, as revealed by R and K responses. The study by Solomon et al., (2019) showed that the semantic and temporal distance of learned words stored in declarative memory is represented in an abstract cognitive map. We showed that in addition to semantic material, phonological distance can be maintained in long-term memory with spatial distance properties. This information might be stored in an allocentric (i.e., object-to-object relations, in this case, the distance between items) *low-dimensional space* (Bottini & Doeller, 2020), where axes are the spatial distance and the phonological similarity of word pairs. It can be that this allocentric *low-dimensional space* is grounded in the perceptual, motor, and introspective states, supporting the notion that phonology is an embodied process (Fogassi & Ferrari, 2008; Gallese & Lakoff, 2005; Nathan, 2017) and that information in *low-dimensional spaces* is rooted in our sensorimotor experience with the body and environment (Barsalou, 2008; Bottini & Doeller, 2020; Wilson, 2002).

Concerning false memories based on semantic features (third hypothesis), phonological similarity/dissimilarity has driven the spatial judgment of (spatially) unlearned item pairs. Indeed, typically phonological false memories occur due to surface similarity between words in a list (Chang & Brainerd, 2021); this perceptual similarity could be used to estimate distances of noun pairs (Boot & Pecher, 2010; Lakoff & Johnson, 1999). This finding suggests that it could be possible that phonological distance is inherently represented using phonological characteristics of stimulus pairs.

Importantly, very low ICC in the LMM highlighted that the proportion of explained variance in the dependent variable is greatly due to the lower levels (i.e., spatial distance and phonology fixed effects) of the model (Monsalves et al., 2020). This strengthens our results in terms of the manipulated independent variables. In addition, Bayesian statistics can overcome the limitations of the frequentist approach and our Bayesian results demonstrate strong to extreme evidence in support of the existence of a phonological distance where words are located depending on their phonological features.

However, this study has several limitations as this is the first attempt to establish a relation between phonology and metaphoric spatial distance. First of all, the lack of neurophysiological data, like in previous studies (e.g., Solomon et al., 2019), could strengthen the findings, which in this work are open only to behavioral and cognitive explanations. Then, we acknowledge that the manipulation of phonology can be improved and that Italian orthography is, in most cases, overlapping with phonology (i.e., transparent grapheme-to-phoneme relationships). In addition, we used alliteration as a marker of phonological similarity, however, other parameters could be used (e.g., biphone or triphone probability, neighborhood size). Furthermore, in some participants, the recognition memory performance was low, and this reduced the number of observations for R and K responses and the related spatial judgments for some participants; this is especially true for the FA R condition (see also 95%CI for the effect size in some results). Moreover, the recognition part required different steps with long instructions (old-new and RK) to be remembered and a bias in recognition memory might be present due to this cognitive load. In addition, it would be interesting to test if phonological distance is affected by the direction of the conceptual mapping (similarity is closeness or closeness is similarity).

Here, we showed that the relation between conceptual similarity and space as a metaphor extends to phonology, in addition to semantic and visual perception domains. This research suggests that, in addition to a physical space, abstract cognitive space is used to represent information and concepts. This abstract space is stored in memory, and it is used to create and understand metaphors.

## Supplementary Information

Below is the link to the electronic supplementary material.

Supplementary file1 (DOCX 23 kb)

## Data Availability

The datasets analyzed during the current study are available in the OSF repository, https://osf.io/9fhac/https://doi.org/10.17605/OSF.IO/9FHAC. The word pairs are available in Supplementary Material 1.

## Code availability

The code used for the statistical part is available in the OSF repository, https://osf.io/9fhac/https://doi.org/10.17605/OSF.IO/9FHAC.
