# Supplementary material for: Phonological similarity between words is represented in declarative memory as spatial distance
Source: Psychol Res. 2023 May 19;87(8):2499–510. doi: 10.1007/s00426-023-01830-y (PMC10197043; doi:10.1007/s00426-023-01830-y)
Supplement: Supplementary file 1 — Supplementary file1 (DOCX 23 kb) [file 426_2023_1830_MOESM1_ESM.docx]

Supplementary Material 1

| Old_fixed | Old_moving | new_fixed | new_moving | Phonology Phonemes |
| --- | --- | --- | --- | --- |
| MA-DRE | MA-NO |  |  | alliterative |
| NO-CE | NO-DO |  |  | alliterative |
| RA-DIO | RA-NA |  |  | alliterative |
| SA-GRA | SA-LE |  |  | alliterative |
| CO-RDA | CO-RPO |  |  | alliterative |
| VI-NO | VI-TA |  |  | alliterative |
| PA-DRE | PA-STA |  |  | alliterative |
| SE-NO | SE-TA |  |  | alliterative |
| CA-RRO | CA-RTA |  |  | alliterative |
| MA-GO | MA-RE |  |  | alliterative |
| PE-PE | PE-SO |  |  | alliterative |
| SE-GNO | SE-TE |  |  | alliterative |
| TE-LA | TE-STO |  |  | alliterative |
| BA-NCA | BA-NDO |  |  | alliterative |
| RO-BA | RO-GO |  |  | alliterative |
| LI-NO | LI-RA |  |  | alliterative |
| MI-RA | MI-TO |  |  | alliterative |
| LO-DE | LO-GO |  |  | alliterative |
| CI-GNO | CI-MA |  |  | alliterative |
| FA-RO | FA-TA |  |  | alliterative |
| BE-NDA | TI-GRE |  |  | dissimilar |
| FA-LCO | GA-MBA |  |  | dissimilar |
| FI-ORE | TO-PO |  |  | dissimilar |
| AR-MA | LA-TTE |  |  | dissimilar |
| LA-DRO | SP-INA |  |  | dissimilar |
| GA-TTO | SO-LE |  |  | dissimilar |
| ST-UFA | UR-LO |  |  | dissimilar |
| FE-RRO | LU-CE |  |  | dissimilar |
| GI-OCO | RA-TTO |  |  | dissimilar |
| MO-NDO | SC-ARPA |  |  | dissimilar |
| PE-STE | FE-LPA |  |  | dissimilar |
| SF-ERA | LE-TTO |  |  | dissimilar |
| SA-NTO | BA-RCA |  |  | dissimilar |
| ME-SE | FI-ENO |  |  | dissimilar |
| SC-ALA | PR-ATO |  |  | dissimilar |
| GU-FO | FI-UME |  |  | dissimilar |
| VA-SO | TE-NDA |  |  | dissimilar |
| VA-SCA | LA-RDO |  |  | dissimilar |
| TR-ENO | PE-SCE |  |  | dissimilar |
| FU-NGO | CO-STA |  |  | dissimilar |
|  |  | BA-GNO | BA-RA | alliterative |
|  |  | CE-NA | CE-STO | alliterative |
|  |  | NA-SO | NA-VE | alliterative |
|  |  | CA-NE | CA-SA | alliterative |
|  |  | TO-RRE | TO-RTA | alliterative |
|  |  | VE-SPA | VE-TRO | alliterative |
|  |  | MU-RO | MU-SA | alliterative |
|  |  | MO-DA | MO-TO | alliterative |
|  |  | SE-ME | SE-RA | alliterative |
|  |  | NO-IA | NO-ME | alliterative |
|  |  | AR-IA | CI-BO | dissimilar |
|  |  | LA-GO | PO-STA | dissimilar |
|  |  | DI-TO | PI-ZZA | dissimilar |
|  |  | AS-SO | CA-LZA | dissimilar |
|  |  | PA-NCA | TA-RLO | dissimilar |
|  |  | MI-ELE | VI-STA | dissimilar |
|  |  | PA-RTE | BA-CO | dissimilar |
|  |  | TE-TTO | PI-EDE | dissimilar |
|  |  | LI-BRO | GO-NNA | dissimilar |
|  |  | PE-NNA | CU-ORE | dissimilar |
